# Association between MetS-IR and prediabetes risk and sex differences: a cohort study based on the Chinese population

**DOI:** 10.3389/fendo.2023.1175988

**Published:** 2023-05-15

**Authors:** Qiyang Xie, Maobin Kuang, Song Lu, Xin Huang, Chao Wang, Shuhua Zhang, Guotai Sheng, Yang Zou

**Affiliations:** ^1^ Department of Cardiology, Jiangxi Provincial People’s Hospital, Medical College of Nanchang University, Nanchang, Jiangxi, China; ^2^ Jiangxi Cardiovascular Research Institute, Jiangxi Provincial People’s Hospital, The First Affiliated Hospital of Nanchang Medical College, Nanchang, Jiangxi, China

**Keywords:** prediabetes, metabolic score for insulin resistance, Chinese, cohort study, METS-IR

## Abstract

**Objective:**

The metabolic score for insulin resistance (MetS-IR) is an emerging surrogate marker for insulin resistance (IR). This study aimed to investigate the association and sex differences between MetS-IR and prediabetes risk in a Chinese population.

**Methods:**

This cohort study included 100,309 adults with normoglycemia at baseline and had followed longitudinally for 5 years, and with prediabetes, defined according to the 2018 American Diabetes Association (ADA) recommended diagnostic criteria, as the outcome of interest. Multivariate Cox proportional hazards regression and restricted cubic spline (RCS) regression models were used to assess the association between MetS-IR and prediabetes risk.

**Results:**

During an observation period of 312,843 person-years, 7,735 (14.84%) men and 4,617 (9.57%) women with pre-diabetes onset were recorded. After fully adjusting for confounders, we found an independent and positive correlation between MetS-IR and the risk of prediabetes in the Chinese population, and the degree of correlation was stronger in women than in men (HR: 1.24 vs 1.16, *P*-interaction<0.05). Furthermore, using RCS nested in the Cox regression model, we found that there was a nonlinear correlation between MetS-IR and prediabetes risk in both sexes with an obvious saturation effect point, and when the MetS-IR was greater than the value of the saturation effect point, the risk of prediabetes was gradually leveling off. We further calculated the saturation effect points of MetS-IR used to evaluate the risk of prediabetes which in men was 42.82, and in women was 41.78.

**Conclusion:**

In this large cohort study, our results supported that MetS-IR was independently and positively associated with the risk of prediabetes in the Chinese population, with the association being stronger in women than in men.

## Introduction

Prediabetes is a transitional state in which the body’s glucose metabolism is between normal and diabetic, including impaired glucose tolerance and impaired fasting glucose (1). The prevalence of prediabetes varies widely worldwide, with recent epidemiological surveys showing a prevalence of approximately 10.0%-40.4% in adults (2–5), and among them, the prevalence of prediabetes in China is about 35.7% (6). It is well known that prediabetes is considered a precursor to diabetes (7), and up to 70% of prediabetes will eventually develop into diabetes (1). In addition, like diabetes, prediabetes can cause damage to multiple organ systems throughout the body (8), including cardiovascular and cerebrovascular, renal, hepatic, and pulmonary systems, and can trigger and exacerbate the risk of developing many chronic diseases (9–12), causing a great economic burden to society and families (13, 14). It is therefore essential to find reliable predictors to identify high-risk groups of prediabetes for early intervention to prevent the progression of abnormal glucose metabolism and the development of complications.

IR is clinically defined as the impaired ability of insulin to uptake and utilize glucose (15) and is an important predisposing factor in many chronic diseases (8). The gold standard for assessing IR is the hyperinsulinemic-euglycemic clamp (16), a complex, time-consuming, and resource-consuming method making it a great challenge for daily clinical application. MetS-IR, a new index for assessing IR, has recently been widely accepted by researchers as a cost-effective predictor of diabetes due to its simple, reliable, and reproducible properties (16). Additionally, observational studies have shown that MetS-IR is not only useful for assessing the risk of diabetes (16–19), but also has excellent risk assessment ability for a variety of metabolism-related diseases such as nonalcoholic fatty liver disease (NAFLD), hyperuricemia, prehypertension, hypertension, and metabolic syndrome (20–25). However, current evidence for the association between MetS-IR and prediabetes risk is still very limited; previous experience suggested that the associations between prediabetes risk and IR was significantly different between the sexes (26, 27). Therefore, this study aimed to retrospectively analyze the association and sex differences between MetS-IR and the risk of prediabetes based on a large cohort of the Chinese population.

## Methods

### Study design and subjects

**Figure 1:**
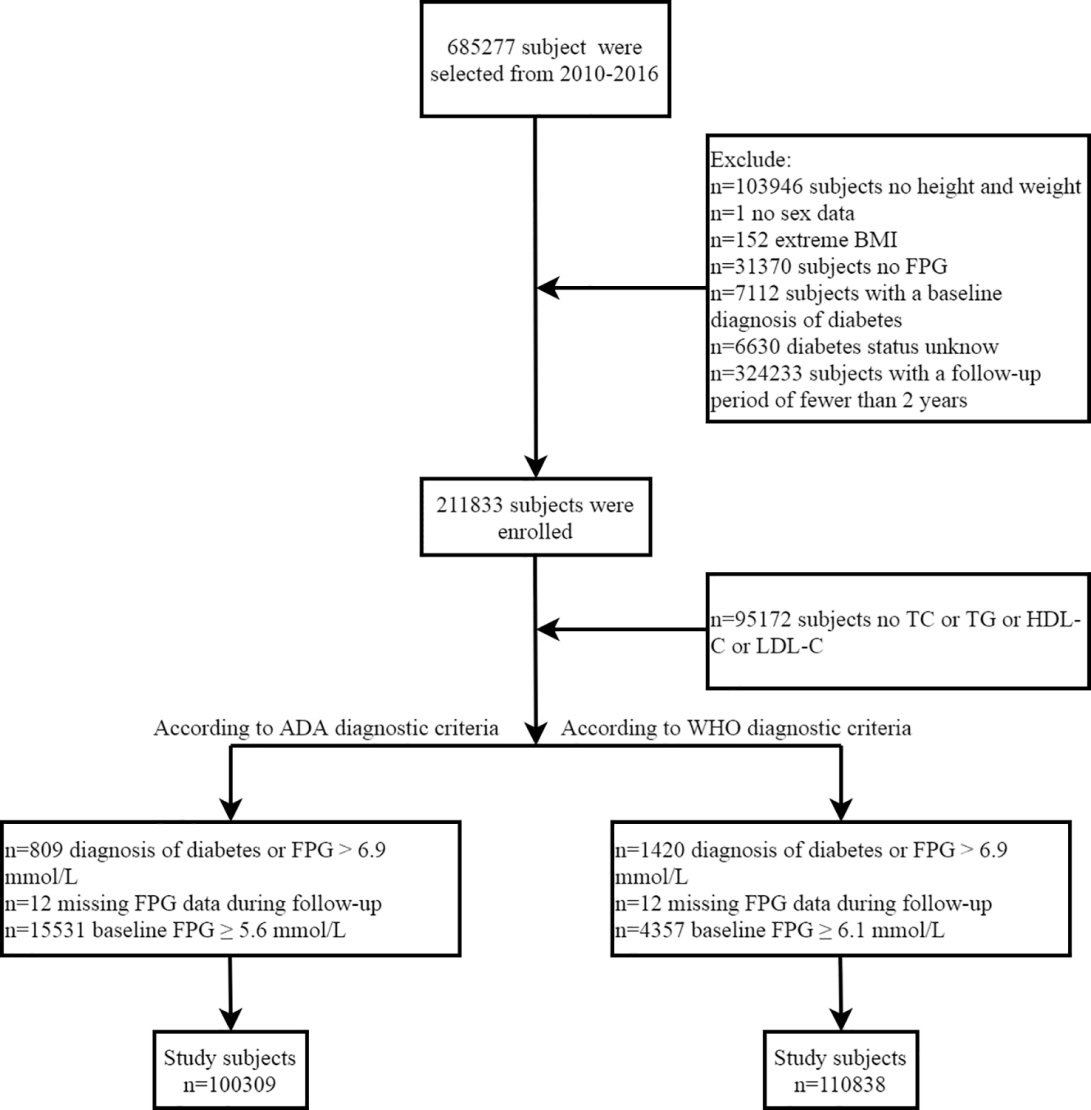
Flowchart of the selection process of study subjects.

The data of this retrospective cohort study comes from the database established by Rich Healthcare Group in China. The available study dataset has been shared in the Dryad public database by Chen et al. (https://datadryad.org/stash/dataset/doi:10.5061%2Fdryad.ft8750v). Under the Dryad public database service rules, all researchers can use the data set in the database for in-depth analysis and to provide new and useful research evidence without infringing on the rights of the dataset’s contributing authors. The retrospective cohort study design has been described in detail previously (28). Overall, subjects were adults recruited at 32 health examination centers in 11 Chinese cities between 2010 and 2016 who received at least two health check-ups during this period. In the original study, Chen et al. analyzed the relationship between body mass index (BMI) and diabetes. They excluded subjects diagnosed with diabetes at baseline, subjects who did not record height, weight, fasting plasma glucose (FPG), and sex information at baseline, subjects with extreme BMI, subjects with a follow-up interval of fewer than 2 years, and subjects whose diabetes status could not be determined during follow-up. This study aimed to further analyze the association between MetS-IR and prediabetes, and based on the previous study design by Chen et al., subjects with the following characteristics were further excluded: (1) FPG ≥ 5.6 mmol/L (ADA criteria) or 6.1 mmol/L (WHO criteria) at baseline (29, 30); (2) missing data for lipid-related parameters; (3) diagnosed diabetes mellitus or FPG > 6.9 mmol/L or missing FPG data during the observation period. Finally, 100,309 and 110,838 subjects were included in this study according to the ADA and WHO recommended diagnostic criteria for prediabetes (**Figure 1**), respectively. The main results of this study were obtained by analyzing the data of subjects screened by the ADA diagnostic criteria, while the data of subjects screened by the WHO diagnostic criteria was used as a sensitivity analysis to verify the reliability of the main analysis results. Given that this study was a secondary analysis of a previous study and that subject-identifying information was anonymized, the Ethics Committee of Jiangxi Provincial People’s Hospital waived informed consent from the subjects and also reviewed and approved the design of this study.

### Measurement of clinical index

When the subjects were interviewed, they were required to fill out a detailed questionnaire, which was used to record relevant demographic data, smoking and drinking information, past medical history, and family history of diabetes. Professional staff measured the subject’s height, weight, and blood pressure (BP) in a quiet environment; when measuring height and weight, participants were required to wear light clothing and take off their shoes and BP was measured with a standard mercury cuff sphygmomanometer. BMI was calculated by dividing the weight (kg) by the height (m) squared. Subjects were required to fast for at least 10 hours before collecting venous blood. After the blood samples were collected and sent for inspection, the medical staff measured the laboratory indicators of the participants on the automatic analyzer (Beckman 5800), including serum triglyceride (TG), total cholesterol (TC), low-density lipoprotein cholesterol (LDL-C), high-density lipoprotein cholesterol (HDL-C), FPG, alanine aminotransferase (ALT), aspartate aminotransferase (AST), blood urea nitrogen (BUN) and creatinine (Cr).

### Diagnosis of prediabetes and calculation of MetS-IR

During the follow-up, according to the 2018 ADA diagnostic criteria for prediabetes, patients with prediabetes were defined as having an FPG between 5.6 and 6.9 mmol/L (29). Similarly, according to the WHO diagnostic criteria for prediabetes, prediabetes patients were defined as having an FPG between 6.1 and 6.9 mmol/L (30). The calculation formula of MetS-IR is as follows: (ln ((2 × FPG) + TG) × BMI)/(ln (HDL-C)) (16).

### Statistical analysis

#### Baseline description

We summarized the baseline characteristics of subjects according to MetS-IR quintiles and whether they were diagnosed with prediabetes. Differences between groups for continuous variables were compared by the Kruskal-Wallis H test or one-way ANOVA, and the chi-square test was chosen for categorical variables.

#### Validation before Cox regression analysis

Before building the Cox proportional hazards regression models, we assessed the feasibility of the modeling. (1) Collinearity diagnosis: the variance inflation factor (VIF) of covariates was calculated by multiple linear regression, and covariates with VIF greater than 5 were considered collinear covariates (**Supplementary Table 1**). (2) Proportional hazards assumption test: to determine whether the proportional hazards assumption was violated by observing whether the Kaplan-Meier curves of prediabetes corresponding to the MetS-IR quintiles constructed in both sexes are crossed.

#### Correlation analysis

According to the STROBE statement (31), we established 4 Cox regression models based on a stepwise adjustment strategy, calculated hazard ratios (HR) and 95% confidence intervals (CI) for the associations between MetS-IR and prediabetes in both sexes, and compare whether there were differences in MetS-IR related prediabetes risks in both sexes through likelihood ratio test, and judged whether there was interaction. Among the 4 models, the crude model did not adjust for any confounding factors; model I adjusted for age, height, family history of diabetes, smoking status and drinking status; model II further considered the influence of blood glucose, blood lipids, and BP based on model I; model III further adjusted for ALT, BUN, and Cr based on model II. In addition, we used the RCS (based on model III, with 5 nodes) nested in the Cox regression model for fitting the shape of the dose-response relationship between MetS-IR and the risk of prediabetes. If the RCS analysis shows that there is a nonlinear association between MetS-IR and prediabetes, the potential threshold or saturation effect point of the nonlinear association will be further calculated by the recursive method through segmental Cox regression.

#### Sensitivity analysis

In addition to performing the same association analysis in subjects included according to WHO criteria, we further considered the effects of BP, lipids, and family history of diabetes on prediabetes (32–34) and performed the same analysis in a population with normal BP, no family history of diabetes, and no hypertriglyceridemia.

## Results

### Baseline characteristics

The study included 100,309 Chinese adults (mean age 42.9 years, 51.97% men and 48.03% women) with normal blood glucose at baseline according to ADA prediabetes diagnostic criteria. **Table 1** shows the baseline characteristics of the subjects grouped by MetS-IR quintiles of both sexes, where the intervals of the men MetS-IR quintiles were Q1 (22.36-37.48), Q2 (37.48-41.79), Q3 (41.79-45.78), Q4 (45.78-50.93) and Q5 (50.93-103.75); while the intervals of women MetS-IR quintiles were Q1 (20.97-32.01), Q2 (32.01-35.14), Q3 (35.14-38.39), Q4 (38.39-43.05), and Q5 (43.05-100.44), respectively. Compared with women, the values corresponding to the MetS-IR quintile intervals were relatively larger in men. In addition, we also found that with increasing MetS-IR quintiles in both sexes, the subjects’ age, height, weight, BMI, SBP, DBP, FPG, TC, TG, LDL-C, ALT, AST, Cr levels and the number with a family history of diabetes gradually increases, while the HDL-C levels gradually decreased (all *P <*0.05).

**Table 1:** Baseline characteristics of subjects grouped according to MetS-IR quintiles for both sexes.

Men						
	MetS-IR quintiles					
	Q1(22.36-37.48)	Q2(37.48-41.79)	Q3(41.79-45.78)	Q4(45.78-50.93)	Q5(50.93-103.75)	P-value
No. of subjects	10426	10426	10426	10426	10426	
Age, years	36.00 (31.00-47.00)	39.00 (33.00-51.00)	41.00 (34.00-52.00)	42.00 (34.00-53.00)	42.00 (35.00-52.00)	<0.001
Height, cm	172.16 (6.22)	171.79 (6.19)	171.65 (6.16)	171.64 (6.19)	171.84 (6.28)	<0.001
Weight, kg	60.06 (6.25)	66.98 (6.03)	71.23 (6.47)	75.34 (7.10)	82.52 (9.72)	<0.001
BMI, kg/m^2^	20.24 (1.58)	22.67 (1.32)	24.15 (1.43)	25.55 (1.62)	27.91 (2.51)	<0.001
SBP, mmHg	117.18 (14.44)	119.86 (14.60)	121.92 (14.81)	123.78 (15.04)	126.36 (15.42)	<0.001
DBP, mmHg	72.61 (9.52)	74.52 (9.94)	76.30 (10.16)	77.94 (10.54)	80.13 (11.07)	<0.001
FPG, mmol/L	4.70 (0.50)	4.78 (0.48)	4.82 (0.47)	4.86 (0.48)	4.89 (0.46)	<0.001
TC, mmol/L	4.56 (0.81	4.68 (0.84)	4.78 (0.86)	4.84 (0.87)	4.95 (0.90)	<0.001
TG, mmol/L	0.84 (0.65-1.10)	1.06 (0.80-1.40)	1.30 (0.97-1.70)	1.56 (1.17-2.10)	2.14 (1.57-2.98)	<0.001
HDL-C, mmol/L	1.51 (0.26)	1.38 (0.23)	1.30 (0.22)	1.21 (0.22)	1.07 (0.22)	<0.001
LDL-C, mmol/L	2.59 (0.61)	2.73 (0.64)	2.81 (0.65)	2.85 (0.65)	2.86 (0.71)	<0.001
ALT, U/L	16.90 (13.00-22.70)	20.00 (15.00-27.50)	23.00 (17.00-32.00)	26.00 (19.00-37.00)	32.00 (22.83-48.00)	<0.001
AST, U/L	21.60 (18.90-25.10)	22.00 (19.00-26.30)	23.20 (20.00-28.00)	24.20 (20.70-29.35)	26.00 (22.00-32.08)	<0.001
BUN, mmol/L	4.90 (1.16)	4.90 (1.14)	4.90 (1.14)	4.91 (1.13)	4.87 (1.12)	0.074
Cr, umol/L	79.45 (11.14)	80.75 (11.60)	80.88 (11.83)	81.21 (12.06) 8	80.69 (12.10)	<0.001
Family history of diabetes						<0.001
	117 (1.12%)	135 (1.29%)	174 (1.67%)	173 (1.66%)	213 (2.04%)	
Smoking status						<0.001
No	974 (9.34%)	885 (8.49%)	985 (9.45%)	1081 (10.37%)	1407 (13.50%)	
Past	159 (1.53%)	215 (2.06%)	225 (2.16%)	248 (2.38%)	233 (2.23%)	
Current	2327 (22.32%)	2258 (21.66%)	2207 (21.17%)	2146 (20.58%)	2144 (20.56%)	
Not recorded	6966 (66.81%)	7068 (67.79%)	7009 (67.23%)	6951 (66.67%)	6642 (63.71%)	
Drinking status						<0.001
No	92 (0.88%)	118 (1.13%)	128 (1.23%)	131 (1.26%)	155 (1.49%)	
Past	735 (7.05%)	840 (8.06%)	880 (8.44%)	907 (8.70%)	900 (8.63%)	
Current	2633 (25.25%)	2400 (23.02%)	2409 (23.11%)	2437 (23.37%)	2729 (26.17%)	
Not recorded	6966 (66.81%)	7068 (67.79%)	7009 (67.23%)	6951 (66.67%)	6642 (63.71%)	
Women						
MetS-IR quintiles						
	Q1(20.97-32.01)	Q2(32.01-35.14)	Q3(35.14-38.39)	Q4(38.39-43.05)	Q5(43.05-100.44)	
No. of subjects	9636	9636	9635	9636	9636	
Age, years	35.00 (30.00-42.00)	37.00 (32.00-45.00)	40.00 (34.00-49.00)	44.00 (35.00-53.00)	48.00 (38.00-59.00)	<0.001
Height, cm	161.47 (5.45)	160.74 (5.46)	160.25 (5.49)	159.60 (5.56)	158.94 (5.79)	<0.001
Weight, kg	48.88 (4.41)	52.76 (4.47)	55.70 (4.79)	59.08 (5.26)	65.89 (7.70)	<0.001
BMI, kg/m^2^	18.73 (1.20)	20.40 (1.11)	21.67 (1.21)	23.17 (1.42)	26.06 (2.52)	<0.001
SBP, mmHg	108.22 (12.89)	110.13 (13.58)	112.44 (14.74)	116.27 (16.14)	123.07 (17.86)	<0.001
DBP, mmHg	68.17 (8.86)	68.85 (9.29)	70.03 (9.74)	71.97 (10.28)	76.00 (11.10)	<0.001
FPG, mmol/L	4.59 (0.48)	4.71 (0.45)	4.77 (0.44)	4.84 (0.43)	4.91 (0.43)	<0.001
TC, mmol/L	4.70 (0.84)	4.62 (0.84)	4.66 (0.88)	4.78 (0.93)	4.91 (0.97)	<0.001
TG, mmol/L	0.67 (0.53-0.85)	0.74 (0.57-0.96)	0.84 (0.63-1.10)	1.00 (0.75-1.37)	1.40 (1.00-2.00)	<0.001
HDL-C, mmol/L	1.74 (0.30)	1.56 (0.26)	1.47 (0.24)	1.38 (0.23)	1.24 (0.24)	<0.001
LDL-C, mmol/L	2.61 (0.62)	2.62 (0.63)	2.68 (0.66)	2.79 (0.70)	2.88 (0.74)	<0.001
ALT, U/L	12.30 (10.00-15.80)	12.60 (10.00-16.40)	13.10 (10.50-17.50)	14.30 (11.20-19.50)	17.30 (13.10-24.20)	<0.001
AST, U/L	19.40 (17.00-22.70)	19.30 (17.00-23.00)	19.80 (17.00-23.00)	20.30 (17.20-24.00)	21.40 (18.00-26.00)	<0.001
BUN, mmol/L	4.25 (1.07)	4.27 (1.08)	4.32 (1.12)	4.42 (1.13)	4.48 (1.14)	<0.001
Cr, umol/L	57.42 (8.58)	57.87 (9.72)	58.09 (10.23)	58.71 (9.58)	58.97 (12.45)	<0.001
Family history of diabetes						<0.001
	221 (2.29%)	250 (2.59%)	295 (3.06%)	312 (3.24%)	318 (3.30%)	
Smoking status						0.082
No	3 (0.03%)	1 (0.01%)	4 (0.04%)	3 (0.03%)	6 (0.06%)	
Past	2 (0.02%)	0 (0.00%)	1 (0.01%)	5 (0.05%)	2 (0.02%)	
Current	1978 (20.53%)	2062 (21.40%)	2057 (21.35%)	1945 (20.18%)	2075 (21.53%)	
Not recorded	7653 (79.42%)	7573 (78.59%)	7573 (78.60%)	7683 (79.73%)	7553 (78.38%)	
Drinking status						0.216
No	3 (0.03%)	4 (0.04%)	3 (0.03%)	3 (0.03%)	2 (0.02%)	
Past	55 (0.57%)	50 (0.52%)	49 (0.51%)	59 (0.61%)	73 (0.76%)	
Current	1925 (19.98%)	2009 (20.85%)	2010 (20.86%)	1891 (19.62%)	2008 (20.84%)	
Not recorded	7653 (79.42%)	7573 (78.59%)	7573 (78.60%)	7683 (79.73%)	7553 (78.38%)	

BMI, body mass index; SBP, systolic blood pressure; DBP, diastolic blood pressure; FPG, fasting plasma glucose; TG, triglyceride; TC, total cholesterol; HDL-C, high-density lipoprotein cholesterol; LDL-C, low-density lipoprotein cholesterol; ALT, alanine aminotransferase; AST, aspartate aminotransferase; BUN, blood urea nitrogen; Cr, creatinine; METS-IR, metabolic score for insulin resistance; Q1, Q2, Q3, Q4and Q5 are quintiles of the MetS-IR.

**Figure 2:**
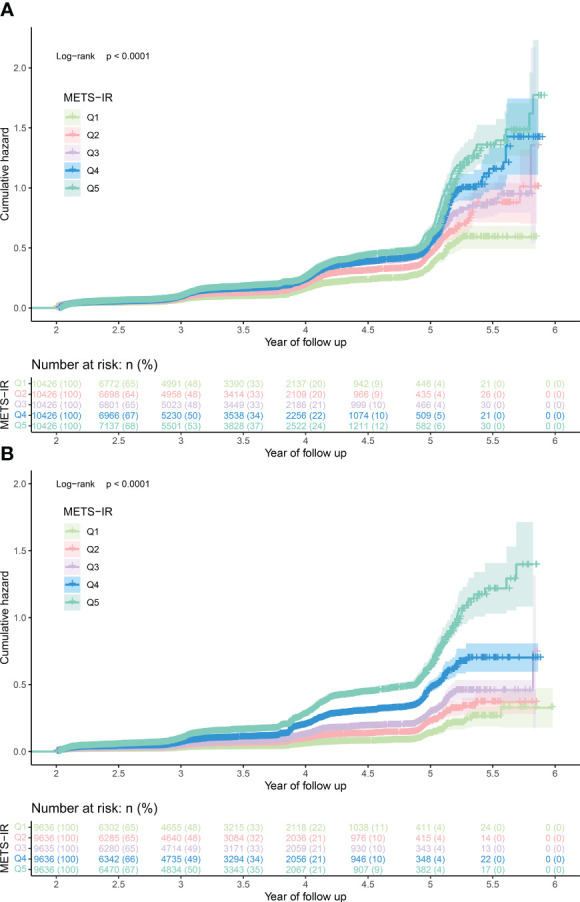
The cumulative hazard of prediabetes among the MetS-IR quintiles in men **(A)** and women **(B)**. MetS-IR, metabolic score for insulin resistance.

During the observation period of 312,843 person-years, the incidence rate of prediabetes was 3948.31/100000 person-years, and the incidence rates of prediabetes in men and women were 4752.84/100000 person-years and 3073.24/100000 person-years, respectively. The Kaplan-Meier curves showed (**Figure 2**) that the cumulative hazard of prediabetes among the MetS-IR quintiles increased gradually without crossing each other, which was consistent with the proportional hazard assumption. Additionally, the covariates weight, BMI, and TC will not be included in the multivariate Cox regression models based on the requirement of VIF for the covariates.

**Table 2** describes the clinical baseline characteristics of both sexes grouped according to whether they developed prediabetes during the observation period. In both sexes, people with prediabetes typically had higher levels of age, weight, BMI, SBP, DBP, Cr, BUN, AST, ALT, LDL-C, TG, TC, and MetS-IR, and a higher proportion of people with a family history of diabetes. In addition, women patients had relatively higher age, TC, and HDL-C, and relatively lower height, weight, BMI, ALT, AST, BUN, and Cr levels compared to men with prediabetes.

**Table 2:** Characteristics of the study participants with and without prediabetes.

	Men			Women		
	Prediabetes	Normoglycemia	*P*-value	Prediabetes	Normoglycemia	*P*-value
No. of subjects	7735	44395	<0.001	4617	43562	<0.001
MetS-IR	46.54 (8.30)	44.07 (8.08)	<0.001	41.63 (7.53)	37.39 (6.75)	<0.001
Age, years	48.26 (13.75)	42.23 (12.33)	<0.001	50.25 (13.22)	41.87 (11.73)	<0.001
Height, cm	171.21 (6.23)	171.92 (6.20)	<0.001	159.14 (5.80)	160.31 (5.59)	<0.001
Weight, kg	73.05 (10.64)	70.91 (10.42)	<0.001	159.14 (5.80)	56.13 (7.80)	<0.001
BMI, kg/m2	24.89 (3.15)	23.97 (3.10)	<0.001	23.53 (3.23)	21.84 (2.88)	<0.001
SBP, mmHg	126.25 (16.57)	121.05 (14.81)	<0.001	123.37 (18.88)	113.04 (15.37)	<0.001
DBP, mmHg	78.95 (11.08)	78.95 (11.08)	<0.001	75.35 (11.51)	70.54 (10.03)	<0.001
FPG, mmol/L	5.04 (0.40)	4.77 (0.48)	<0.001	5.02 (0.41)	4.74 (0.46)	<0.001
TC, mmol/L	4.88 (0.87)	4.74 (0.86)	<0.001	4.98 (0.96)	4.71 (0.89)	<0.001
TG, mmol/L	1.46 (1.00-2.11)	1.25 (0.89-1.83)	<0.001	1.10 (0.76-1.60)	0.85 (0.62-1.20)	<0.001
HDL-C, mmol/L	1.29 (0.28)	1.29 (0.27)	0.693	1.43 (0.29)	1.48 (0.31)	<0.001
LDL-C, mmol/L	2.82 (0.65)	2.76 (0.66)	<0.001	2.88 (0.70)	2.70 (0.67)	<0.001
ALT, U/L	24.40 (17.58-36.00)	22.50 (16.20-33.00)	<0.001	16.00 (12.00-22.00)	13.60 (10.80-18.10)	<0.001
AST, U/L	24.00 (20.00-29.00)	23.00 (20.00-28.00)	<0.001	21.00 (18.00-25.00)	20.00 (17.00-23.50)	<0.001
BUN, mmol/L	5.00 (1.15)	4.88 (1.13)	<0.001	4.56 (1.13)	4.32 (1.11)	<0.001
Cr, umol/L	81.05 (12.40)	80.51 (11.65)	<0.001	59.78 (10.55)	58.05 (10.15)	<0.001
Family history of diabetes			<0.001			0.448
	168 (2.17%)	644 (1.45%)		142 (3.08%)	1254 (2.88%)	
Smoking status			<0.001			0.010
No	835 (10.80%)	4497 (10.13%)		5 (0.11%)	12 (0.03%)	
Past	155 (2.00%)	925 (2.08%)		0 (0.00%)	10 (0.02%)	
Current	1395 (18.03%)	9687 (21.82%)		927 (20.08%)	9190 (21.10%)	
Not recorded	5350 (69.17%)	29286 (65.97%)		3685 (79.81%)	34350 (78.85%)	
Drinking status			<0.001			0.433
No	98 (1.27%)	526 (1.18%		2 (0.04%)	13 (0.03%)	
Past	576 (7.45%)	3686 (8.30%)		28 (0.61%)	258 (0.59%)	
Current	1711 (22.12%)	10897 (24.55%)		902 (19.54%)	8941 (20.52%)	
Not recorded	5350 (69.17%)	29286 (65.97%)		3685 (79.81%)	34350 (78.85%)	

BMI, body mass index; SBP, systolic blood pressure; DBP, diastolic blood pressure; FPG fasting plasma glucose; TG, triglyceride; TC, total cholesterol; HDL-C, high-density lipoprotein cholesterol; LDL-C, low-density lipoprotein cholesterol; ALT, alanine aminotransferase; AST, aspartate aminotransferase; BUN, blood urea nitrogen; Cr, creatinine; MetS-IR, metabolic score for insulin resistance.

### Association of MetS-IR with prediabetes in both sexes

Four Cox regression models were developed to investigate the association of MetS-IR with prediabetes and sex differences (**Table 3**). In the crude model, MetS-IR was positively associated with prediabetes in both sexes. However, in the models further adjusted for confounding factors (models I-III), the positive association between MetS-IR and prediabetes remained unchanged, and the degree of association was slightly weakened. In model III, each standard deviation increase in MetS-IR was associated with a 16% increased risk of prediabetes in men (HR: 1.16, 95%CI 1.12, 1.20, *P*-trend < 0.001), and each increase in MetS-IR standard deviation in women, the risk of prediabetes increased by 24% (HR: 1.24, 95%CI 1.19, 1.35, *P*-trend < 0.001). Additionally, the interaction test suggested that the risk of prediabetes associated with MetS-IR in women was higher than that in men (*P*-interaction < 0.0001).

**Table 3:** Cox regression analyses for the association between MetS-IR and the incidence of prediabetes.

	Hazard ratios (95% confidence interval)				
	Crude model	Model I	Model II	Model III	*P*-interaction
Sex					<0.0001
Men					
MetS-IR (per SD increase)	1.21 (1.18, 1.23)	1.19 (1.16, 1.21)	1.19 (1.15, 1.23)	1.16 (1.12, 1.20)	
MetS-IR (Quintiles)					
Quintile 1	Ref	Ref	Ref	Ref	
Quintile 2	1.31 (1.20, 1.42)	1.24 (1.14, 1.35)	1.24 (1.14, 1.35)	1.21 (1.11, 1.32)	
Quintile 3	1.59 (1.47, 1.73)	1.48 (1.37, 1.60)	1.46 (1.34, 1.59)	1.41 (1.29, 1.54)	
Quintile 4	1.68 (1.55, 1.82)	1.54 (1.42, 1.66)	1.46 (1.34, 1.60)	1.38 (1.26, 1.52)	
Quintile 5	1.92 (1.78, 2.07)	1.77 (1.64, 1.90)	1.70 (1.53, 1.87)	1.57 (1.42, 1.74)	
*P*-trend	<0.0001	<0.0001	<0.0001	<0.0001	
Women					
MetS-IR (per SD increase)	1.51 (1.48, 1.54)	1.35 (1.31, 1.38)	1.24 (1.20, 1.29)	1.24 (1.19, 1.29)	
MetS-IR (Quintiles)					
Quintile 1	Ref	Ref	Ref	Ref	
Quintile 2	1.72 (1.50, 1.96)	1.56 (1.37, 1.79)	1.43 (1.25, 1.64)	1.42 (1.23, 1.63)	
Quintile 3	2.25 (1.98, 2.56)	1.88 (1.66, 2.14)	1.67 (1.46, 1.92)	1.67 (1.46, 1.92)	
Quintile 4	3.52 (3.12, 3.98)	2.62 (2.32, 2.96)	2.19 (1.91, 2.50)	2.20 (1.92, 2.52)	
Quintile 5	5.19 (4.62, 5.83)	3.38 (3.00, 3.81)	2.52 (2.18, 2.92)	2.49 (2.15, 2.88)	
*P*-trend	<0.0001	<0.0001	<0.0001	<0.0001	

Crude model adjusted for none.

Model I adjusted for age, height, family history of diabetes, smoking status and drinking status.

Model II adjusted for age, height, family history of diabetes, smoking status, drinking status, SBP, DBP, FPG, TG, HDL-C and LDL-C.

Model III adjusted for age, height, family history of diabetes, smoking status, drinking status, SBP, DBP, FPG, TG, HDL-C, LDL-C, ALT, BUN and Cr.

### Nonlinear analysis

**Figure 3:**
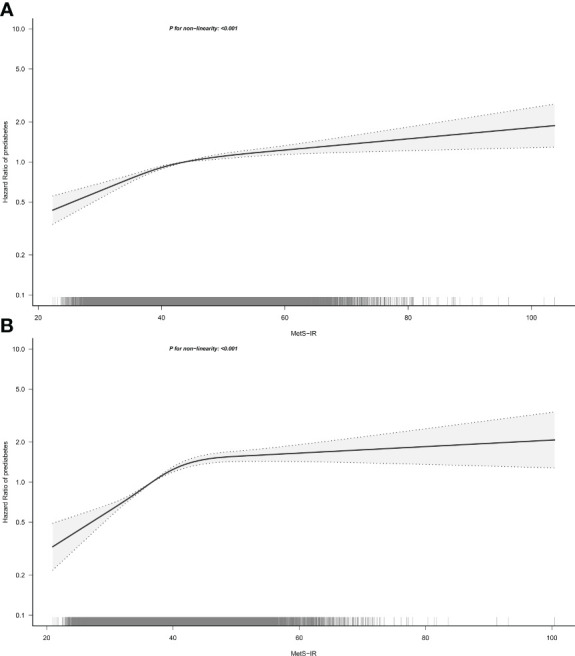
Hazard ratios for the nonlinear relationship between MetS-IR and the risk of prediabetes in men **(A)** and women **(B)**. MetS-IR, metabolic score for insulin resistance. Adjusted for age, height, family history of diabetes, smoking status, drinking status, SBP, DBP, FPG, TG, HDL-C, LDL-C, ALT, BUN and Cr.

To further analyze the association between MetS-IR and the risk of prediabetes in both sexes, we fitted dose-response relationship curves for the association using RCS. As shown in **Figure 3**, the correlation between MetS-IR and prediabetes risk of both sexes was positive and nonlinear with an obvious saturation effect point, and when the MetS-IR was greater than the value of the saturation effect point, the risk of prediabetes was gradually leveling off. We further calculated the saturation effect points of MetS-IR used to evaluate the risk of prediabetes which in men was 42.82, and in women was 41.78 (**Table 4**).

**Table 4:** Thresholds for predicted MetS-IR-related prediabetes risk.

	Prediabetes (HR, 95% CI)	
	Men	Women
Fitting model by multivariable Cox regression		
	1.02 (1.01, 1.02)	1.03 (1.03, 1.04)
Fitting model by two-piecewise Cox regression		
The best inflection point	42.82	41.78
< inflection point	1.04 (1.03, 1.05) <0.0001	1.07 (1.06, 1.09) <0.0001
> inflection point	1.01 (1.01, 1.02) <0.0001	1.01 (1.00, 1.02) 0.0240
*P* for the log-likelihood ratio test	<0.001	<0.001

HR, hazard ratios; CI, confidence interval; other abbreviations as in **Table 1**.

Adjusted for age, height, family history of diabetes, smoking status, drinking status, SBP, DBP, FPG, TG, HDL-C, LDL-C, ALT, BUN and Cr.

### Sensitivity analysis

To further demonstrate the stability of the results, we performed the following four sensitivity analyses: Sensitivity-1: subjects included under the standard definition according to WHO. Sensitivity-2: subjects with no family history of diabetes were retained. Sensitivity-3: excluded subjects with abnormal BP levels. Sensitivity-4: excluded subjects with abnormal TG levels. In the multiple sensitivity analysis (**Table 5**), all results were in general agreement with the main analysis, indicating the strong robustness of our results.

**Table 5:** Adjusted hazard ratios and 95% confidence intervals for prediabetes risk associated with the MetS-IR in different test populations: sensitivity analysis.

			MetS-IR quintiles							
	No.of subjects	MetS-IR (per SD increase)		Q1	Q2	Q3	Q4	Q5	*P*-trend	
Men									
Sensitivity-1	58722	1.35 (1.27, 1.42)		Ref	1.54(1.15,2.06)	2.30(1.75,3.02)	2.80(2.13,3.69)	3.36(2.52,4.48)	<0.0001	
Sensitivity-2	50303	1.16 (1.12, 1.20)		Ref	1.12(0.98,1.26)	1.29(1.15,1.45)	1.45(1.29,1.63)	1.61(1.42,1.83)	<0.0001	
Sensitivity-3	48392	1.20 (1.16, 1.25)		Ref	1.14(1.00,1.29)	1.38(1.22,1.55)	1.57(1.40,1.78)	1.76(1.55,2.00)	<0.0001	
Sensitivity-4	35190	1.16 (1.11, 1.21)		Ref	1.08(0.95,1.22)	1.24(1.10,1.40)	1.32(1.16,1.49)	1.45(1.25,1.66)	<0.0001	
Women										
Sensitivity-1	52116	1.41 (1.31, 1.52)		Ref	1.62(1.30,2.03)	2.36(1.89,2.94)	2.84(2.25,3.58)	3.57(2.76,4.62)	<0.0001	
Sensitivity-2	45214	1.29 (1.23, 1.35)		Ref	1.31(1.18,1.46)	1.82(1.63,2.03)	2.00(1.77,2.26)	2.07(1.80,2.39)	<0.0001	
Sensitivity-3	45442	1.36 (1.30, 1.43)		Ref	1.37(1.23,1.52)	1.90(1.70,2.12)	2.14(1.89,2.41)	2.36(2.05,2.73)	<0.0001	
Sensitivity-4	41359	1.31 (1.25, 1.38)		Ref	1.26(1.13,1.41)	1.69(1.51,1.89)	1.81(1.59,2.06)	1.98(1.69,2.31)	<0.0001	

MetS-IR, metabolic score for insulin resistance.

(1) Sensitivity-1: including 110,838 subjects according to WHO’s diagnostic criteria for prediabetes, adjusted for age, height, family history of diabetes, smoking status, drinking status, SBP, DBP, FPG, TG, HDL-C, LDL-C, ALT, BUN and Cr; (2) sensitivity-2: excluding subjects with a family history of diabetes, adjusted for age, height, smoking status, drinking status, SBP, DBP, FPG, TG, HDL-C, LDL-C, ALT, BUN and Cr.; (3) sensitivity-3: excluding subjects whose SBP ≥ 140 mmHg or DBP ≥ 90 mmHg, adjusted for age, height, family history of diabetes, smoking status, drinking status, FPG, TG, HDL-C, LDL-C, ALT, BUN and Cr.; (4) sensitivity-4: excluding subjects whose TG ≥1.7mmol/L, adjusted for age, height, family history of diabetes, smoking status, drinking status, SBP, DBP, FPG, HDL-C, LDL-C, ALT, BUN and Cr.

## Discussion

In this large longitudinal cohort study based on a Chinese population, our results supported an independently positive association between MetS-IR and the risk of prediabetes, with the magnitude of association stronger in women than in men. To our knowledge, this study is the first to discover a positive correlation between MetS-IR and prediabetes risk in Chinese adults.

The main pathophysiological changes in prediabetes are IR and early beta-cell failure (35). Like diabetes, prediabetes can also cause a variety of microvascular, macrovascular, and internal organ complications (1, 8–12). It is well known that cardiovascular disease is the main cause of fatal events in patients with diabetes (36), and related studies have reported that the risk of future cardiovascular disease can be predicted already in the pre-diabetic period (37, 38). The impact of prediabetes on the health of the general population is significant and, more disturbingly, and there is still a lack of adequate public awareness of prediabetes (39), which represents a huge challenge for the public health of society.

The hyperinsulinemic-euglycemic clamp is recognized as the gold standard for evaluating IR, but due to the relatively complicated measurement method, it cannot be widely used in large-scale epidemiological investigations; While MetS-IR, as a simple IR surrogate indicator, is considered to be widely used (16). The MetS-IR is a new score to assess cardiometabolic risk in healthy and high-risk subjects and a promising tool to screen for insulin sensitivity, as described by Bello-ChavollaOY et al. The value of this parameter has been confirmed in a large number of subsequent epidemiological investigations, including risk assessment and prediction of diabetes, NAFLD, hyperuricemia, prehypertension, and hypertension, hypertension combined with hyperuricemia, metabolic syndrome, and various chronic disease-related complications (16–25, 40). Also, it is worth noting that in a recent study by Li et al., who included 1,205 baseline normoglycemic subjects and evaluated the association between MetS-IR and prediabetes for the first time, their results showed that, MetS-IR was not significantly associated with prediabetes (17). And in the current study, based on a longitudinal cohort of more than 100, 000 ordinary people, we found that there was a positive correlation between MetS-IR and prediabetes. After fully controlling the influence of covariates, this correlation was still stable even in different populations. In addition to this, we found that the risk of prediabetes associated with MetS-IR was significantly higher in women than in men. Compared with the research results of Li et al. (17), the current study believed that there was a longitudinal correlation between MetS-IR and prediabetes, and there were obvious sex differences. For the results of Li et al. who did not find an association between MetS-IR and prediabetes, we consider the following reasons: (1) The relatively small sample size resulted in insufficient statistical power. (2) Combined with some results of the current study, TC and BMI were collinear variables of MetS-IR and should not be directly included in the multivariate Cox regression models for analysis. Therefore, based on the large sample size and strict statistical adjustment strategy of the current study, the relationship between MetS-IR and prediabetes can be considered to be longitudinally relevant.

The sex differences regarding the association between MetS-IR and prediabetes found in this study may be partially explained by the following two reasons. First, the main reason may be related to the fact that the majority of women diagnosed with prediabetes in this study were postmenopausal women (about 70% of women were over 50 years old). It is well known that estrogen is an important regulator of IR, and postmenopausal women are more prone to lipid disorders due to the lack of estrogen (41, 42), which in turn causes central fat deposition and abdominal obesity in postmenopausal women (43–45), and harmful lipid deposition promotes the development of prediabetes. Additionally, the literature reported that women are inherently more prone to IR, but sex hormones, environment, and good lifestyle factors can improve the “genetic disadvantage” of women (46). However, with aging, these “protective factors” gradually lose their effect, and the exposed “genetic disadvantage” makes women more likely to develop IR and develop prediabetes. Second, there are differences in body composition and physical activity levels between the sexes; women generally have higher fat mass and are less physically active than men (47, 48). Fat accumulation is known to cause deleterious effects on glucose metabolism and promote the development of IR in non-adipose tissue organs (49), and women generally have a relatively higher fat mass which may increase the risk of prediabetes. Furthermore, physical activity can promote the process of glucose metabolism, including increasing capillary perfusion to promote muscle glucose uptake, and increasing GLUT4 content in cell membranes to promote glucose transport from interstitium to muscle sarcolemma and t-tubules (50). Therefore, the relative lack of physical activity in women may inhibit the active process of glucose metabolism, thereby increasing the risk of prediabetes.

In the current study, we have identified a linear association between MetS-IR and prediabetes based on multivariate Cox regression models, a finding that was consistent with previous epidemiological studies (17). It should be noted, however, that the linear relationship assessed by Cox regression is more indicative of an overall positive relationship between MetS-IR and prediabetes, while some subtle changes cannot be judged. Therefore, we further fitted the dose-response relationship curves by RCS. As can be seen from **Figure 3**, there was indeed a positive correlation between MetS-IR and prediabetes and a saturation effect point for the correlation. When the MetS-IR in men was greater than 42.82 or the MetS-IR in women was greater than 41.78, the risk of prediabetes almost did not increase. Similar findings were also reported in the previous study by Cai et al. (20), who studied the relationship between MetS-IR and NAFLD in non-obese people and finally found that there was an obvious saturation effect point between MetS-IR and NAFLD, which was calculated to be 36. These new findings based on nonlinear analysis may more accurately show the actual relationship between MetS-IR and prediabetes, and nonlinear analysis should be applied in more association analyses.

The findings of this study provided new evidence for the prevention of prediabetes. Previous experience suggested that a good lifestyle and appropriate use of lipid-lowering drugs are effective strategies to regulate lipid metabolism disorders (51), while the treatment of obesity mainly relies on lifestyle changes, anti-obesity drugs and even bariatric surgery (52). Additionally, the consumption of low-energy foods (such as fresh vegetables, fruits, seaweed, etc.) has a protective effect on lipid metabolism disorders, especially for postmenopausal women (44). Therefore, the present study may provide some suggestions for the prevention of prediabetes: (1) Based on the present findings, a simple indicator, MetS-IR, may be selected to screen people at high risk of prediabetes, especially women. (2) Individuals with high MetS-IR calculated in clinical or physical examinations can be appropriately intervened. We recommend that they should change their irrational lifestyle and try to choose a low-energy diet as well as take the necessary medications to prevent the development of prediabetes (53).

### Study strengths and limitations

Strengths of this study: (1) This study is the first to discover a positive association between MetS-IR and prediabetes and to confirm that there were sex differences in the association, findings that further enriched the understanding of risk factors for prediabetes. (2) The study population covers different regions and age groups in China and has a large sample size, with a good representation of the Chinese population. (3) A series of sensitivity analyses were conducted in this study to emphasize the robustness of the main results. (4) In this study, a nonlinear relationship between MetS-IR and prediabetes was found for the first time, and the potential saturation effect points were calculated. These findings may provide useful evidence for the prevention of prediabetes.

Limitations: (1) The present study relied only on FPG for the diagnosis of prediabetes, which may have underestimated the true prevalence of prediabetes; however, the present study still found a correlation between MetS-IR and the risk of prediabetes, which in fact rather further confirms the reliability of the findings of the present study. (2) Due to this study is a retrospective observational study, the covariates collected are relatively limited, so some important risk/protective factors may have not been evaluated, and there is a certain residual confounding. (3) Although the subjects in the current study come from different cities (Beijing, Shanghai, Nanjing, Suzhou, Changzhou, Nantong, Hefei, Wuhan, Chengdu, Guangzhou, and Shenzhen), all of these cities are relatively developed regions and are southern cities except for Beijing. Therefore, further studies on the applicability of MetS-IR in northern and rural populations may be needed. (4) The follow-up time of this study is short, and prospective studies with a longer follow-up time are needed to verify the stability of the current research results. (5) It should be noted that the smoking and drinking status of many subjects in the current study was not recorded. Since smoking and drinking are potential confounding factors, the missing data on these two variables may affect the control of confounding bias, which may have some influence on the research results.

## Conclusion

In this longitudinal study based on a large sample population in China, we found that MetS-IR was positively and independently associated with the risk of prediabetes; furthermore, women were at higher risk of MetS-IR-related prediabetes compared to men. These new insights may provide a useful reference for the primary prevention of prediabetes and can guide individuals at high risk for abnormal blood glucose metabolism to reduce the risk of developing prediabetes by improving behavior and lifestyle at an early stage.

## Data availability statement

The datasets presented in this study can be found in online repositories. The names of the repository/repositories and accession number(s) can be found in the article/**Supplementary Material**.

## Ethics statement

The studies involving human participants were reviewed and approved by the Ethics Committee of Jiangxi Provincial People’s Hospital. Written informed consent for participation was not required for this study in accordance with the national legislation and the institutional requirements.

## Author contributions

YZ made the study design. All authors conducted the study. QX, MK and SL analyzed the data and wrote the manuscript. YZ attended the manuscript revision. All authors agreed with the final manuscript.
