# Supplementary material for: Association between MetS-IR and prediabetes risk and sex differences: a cohort study based on the Chinese population
Source: Front Endocrinol (Lausanne). 2023 May 15;14:1175988. doi: 10.3389/fendo.2023.1175988 (PMC10226663; doi:10.3389/fendo.2023.1175988)
Supplement: Supplementary file 1 [file Table_1.docx]

Supplementary Table 1: Collinearity diagnostics steps.

|  | Variance inflation factor | | | |
| --- | --- | --- | --- | --- |
|  | Step 1 | Step 2 | Step 3 | Step 4 |
| MetS-IR | 45.5 | 45 | 2.8 | 2.8 |
| Age | 1.3 | 1.3 | 1.3 | 1.3 |
| Sex | 3.5 | 3.4 | 3.4 | 3.4 |
| Height | 54.3 | 2 | 2 | 2 |
| Weight | 180.5 | NA | NA | NA |
| BMI | 119.2 | 24.1 | NA | NA |
| SBP | 2 | 2 | 2 | 2 |
| DBP | 2 | 2 | 2 | 2 |
| FPG | 1.1 | 1.1 | 1.1 | 1.1 |
| TC | 6.6 | 6.6 | 6.6 | NA |
| TG | 3.6 | 3.6 | 1.9 | 1.5 |
| HDL | 7 | 7 | 2.1 | 1.8 |
| LDL | 5.5 | 5.5 | 5.5 | 1.1 |
| ALT | 3.4 | 3.4 | 3.4 | 3.4 |
| AST | 3 | 3 | 3 | 3 |
| BUN | 1.2 | 1.2 | 1.2 | 1.2 |
| Cr | 2.2 | 2.2 | 2.2 | 2.2 |
| Family history of diabetes | 1.3 | 1.3 | 1.3 | 1.3 |
| Smoking status | 1.2 | 1.2 | 1.2 | 1.2 |
| Drinking status | 1 | 1 | 1 | 1 |

Note-1: Variance inflation factor = 1/(1-R^2^). Abbreviations as in Table 1.

Note-2: The variables with Variance inflation factor >5 will be regarded as collinear variables and cannot be included in the multiple regression model.
